# Challenges regarding the control of environmental sources of contamination in healthcare settings in low-and middle-income countries - a narrative review

**DOI:** 10.1186/s13756-020-00747-0

**Published:** 2020-06-09

**Authors:** Folasade T. Ogunsola, Shaheen Mehtar

**Affiliations:** 1 College of Medicine, University of Lagos, Ishaga, Lagos, PMB 12003 Nigeria; 2 Infection Control Africa Network, Cape Town, South Africa; 3 Stellenbosch University Cape Town, Cape Town, South Africa

**Keywords:** Challenges, Environment, Contamination, Infection control, Low-and-lower middle income

## Abstract

**Background:**

Healthcare-associated infections (HAI) especially outbreaks of multi-drug-resistant organisms within hospitals are recognized as a major contributor to morbidity and mortality of hospitalized patients. The healthcare environment can act as an amplifier of HAI during outbreaks. The risk of acquiring HAI are 20 times higher in Low-and-middle-income countries.

The purpose of this article is to review the challenges associated with controlling environmental contamination in low and lower-middle income countries (LMIC), highlighting possible solutions.

**Method:**

This is a narrative review. A literature search was carried out in Google scholar, PubMed, Science Direct, EBSCOHOST, CENGAGE, Scopus, ProQuest, Clinical Key and African journals online using the key words - Health care Associated Infections (HCAIs) in LMICs, Challenges of HAIs in LMIC, Challenges of Prevention and Control of HAIs in LMICs, Environment of care and infection transmission, Contaminated environment and HAIs.

**Results:**

From the accessed databases, 1872 articles related to environmental sources of contamination in healthcare settings were found. Of these, only 530 articles focused on LMICs. However, only 186 articles met the inclusion criteria studies published in English, conducted between 2000 and 2019 and exploring environmental sources of contamination in LMIC healthcare settings). The sources of environmental contamination in healthcare are numerous and commonly associated with poor governance, Inadequate infrastructure, human capacity and inadequate funding. Low awareness exists at all levels as to the role of the environment in healthcare outcomes and may explain in part the low priority given for funding.

**Conclusion:**

Leadership and trained personnel, both Infection prevention and control practitioners and cleaners are crucial to drive and sustain the process to reduce environmental contamination in healthcare environments.

## Introduction

The hospital environment plays an important role in infection transmission particularly for multi-drug-resistant organisms (MDROs) which have the ability to persist in the healthcare environment [1, 2]. This has led to increased recognition of the environment as an amplifier of healthcare associated infections (HAI) particularly during outbreaks of MDROs. The main goal of an infection prevention and control (IPC) program is to reduce risk of healthcare associated infections (HAI) and this includes the healthcare environment. However, the contribution of the healthcare environment in low to middle income countries (LMICs) is largely unknown. The stratification of income groups by The World Bank generally reflects the funding of healthcare budgets of individual countries. Thus, LMIC countries spend less on healthcare service provision and delivery with significant negative impact on clean and safe environments of care [3, 4].

### The significance of the environment in contamination

The environment of care is the space around a patient or healthcare worker where direct or indirect contact occurs by those delivering healthcare. It comprises of three elements; the physical space or building used for providing patient care; equipment used in providing patient care or used within operational procedures of patient care; and people, including healthcare staff and visitors [5]. This physical space during in-patient care can be broadly categorized into ‘high-touch’ and ‘low-touch’ surfaces [6]. The former relates to frequently touched surfaces and therefore a higher risk of HAI transmission, while the latter relates to areas with minimal hand contact and therefore less likely to be a source of transmission [7]. The impact of the environment on infection rates has been demonstrated. When adequate environmental cleaning is lacking, the risk of infection increases when a patient is admitted immediately into an environment previously occupied by another patient infected by the same pathogen [2] thus highlighting the environment as a route of transmission of HAI. This has led to increased attention to environmental cleaning and cleanliness by healthcare systems, in a bid to strengthen IPC measures.

In 2003, the United States Centres for Disease Control and Prevention (CDC) emphasized the importance of implementing proper standards for environmental hygiene, including air, ventilation, water in the built environment [8]. A World Health Organisation (WHO) guideline on the core components of IPC (2016a) emphasized the importance of undertaking patient care activities in a hygienic environment in the prevention and control of HAIs and antimicrobial resistance (AMR). Important recommendations for Water Sanitation and Hygiene (WASH) services, infrastructure as well as the availability of appropriate infection prevention and control (IPC) materials and equipment are included in the implementation guide [9]. In 2019, the Infection Control Africa Network (ICAN), in collaboration with CDC, produced a set of recommendations for LMICs on best practices in environmental cleaning in resource limited settings [10].

The environmental contaminants of healthcare spaces include skin squames, microorganisms and dust. Humans, especially those that are colonized, shed microorganisms into the environment particularly after physical activity [11, 12]. The ability of microorganisms to survive in that environment will depend on temperature and humidity [13], the presence of organic matter capable of supporting the growth of pathogenic microorganisms [14] and the adequacy of cleaning. In healthcare, the rate of removal and dilution of airborne microorganisms, as well as surface characteristics and orientation (horizontal or vertical) have an effect on the survival of microbes [15]. Gram-positive organisms thrive in dry conditions and on dusty surfaces [16, 17], Gram-negative organisms generally persist in damp or moderately humid conditions [18] while fungi flourish not only in damp areas and on fibrous materials but can also survive on dusty surfaces [16]. Many pathogens can survive and retain their infectious potential in the environment for many hours, days or months [2, 19] therefore, in health systems where environmental cleaning is inadequate, the potential risk for HAI are considerably increased.

## Methods

A narrative review of the challenges associated with control of environmental contamination was done. A literature search in Google scholar, PubMed, Science Direct, EBSCOHOST, CENGAGE, Scopus, ProQuest, Clinical Key and African journals online using the key words - Health care Associated Infections (HCAIs) in LMICs, Challenges of HAIs in LMIC, Challenges of Prevention and Control of HAIs in LMICs, Environment of care and infection transmission, Contaminated environment and HAIs. Only articles published in English and between the years 2000–2019 were included. Literature focusing on children, adults, healthcare workers, and the hospital environment were reviewed. Abstracts and titles were evaluated for relevance. Guidelines on environmental cleaning, healthcare associated infections and infection control were also consulted. Practices of hygiene in hospital settings not focusing on LMICs were also used to make comparisons. Publications focusing on guidelines and practices of hospital-environment cleaning, disinfection of healthcare facilities and equipment, structural design guidelines and practices, environmental control systems, supply of portable water, and hospital waste management practices, were reviewed. The reviewed papers highlight the salient features of the challenges faced when implementing effective hospital hygiene and environmental cleaning and disinfection, control systems and practices in LMICs and proffers pragmatic solutions to prevent environmental contamination.

## Results

From the accessed databases, 1872 articles related to environmental sources of contamination in healthcare settings were found. Of these, only 530 articles focused on LMICs. However, only 186 articles met the inclusion criteria studies published in English, conducted between 2000 and 2019 and exploring environmental sources of contamination in LMIC healthcare settings). The review of the relevant studies that met the stated inclusion criteria is presented in the following themes: Structure and Infrastructure; human capacity development; clinical and corporate governance; challenges implementing effective processes, procedures and protocols; and funding of environmental cleaning programme.

### Structure and infrastructure

#### Structural design

Healthcare facilities in LMIC’s are often not purpose-built and may have undergone extensive changes in layout and clinical activity over the years. Surfaces are often covered with materials that are not appropriate for ensuring safety. All health care work surface should be resistant to chemicals used daily for cleaning such as detergents (or disinfectants as appropriate) and can be easily wiped down [20] however some basic materials used in construction and surfaces in LMIC healthcare facilities do not meet international environmental hygiene safety standards [20, 21]. More importantly, these surfaces become damaged due either to the overuse of chemical disinfectants such as chlorine, or a lack of resources for maintenance and repair, till it becomes impossible to maintain a clean and intact environment. The introduction of vaporized hydrogen peroxide or ultraviolet light, following terminal cleaning, have also been proposed as newer ‘non-touch’ (automated) decontamination technologies, however these are either unavailable or unaffordable in most LMICs.(20).

Other structural challenges are inadequate ventilation, inadequate numbers of wards, sinks, width of corridors and walkways, location of patient ablution facilities and inadequate sluice rooms. These often reducing accessibility to adequate cleaning. Ventilation, whether natural or mechanical [22], should be included in the design of purpose-built facilities or during renovations. While natural ventilation can remove and dilute airborne microbes such as *M. tuberculosis* [23], it is not a substitute for good environmental cleaning. High concentrations of airborne bacteria have been reported in densely populated locations such as the pharmacy, lobby and other areas with densities of 0.5 to 1 cfu per m^− 2^ of air in ward areas and these concentrations increased in proportion to the number of people in the room [24, 25] resulting in a heavier bioburden.

#### Overcrowding in wards

The ever-increasing demand on limited hospital beds has a major impact on environmental cleaning. The number of patients admitted in healthcare facilities in many LMICs often exceeds the number of available beds resulting in the sharing of beds or placing of patients on the floors in surrounding hallways and stairwells [26]. Overcrowding contributes to the inability to clean the environment satisfactorily and most importantly, beds are not vacated long enough to clean them properly, change linen and repair or discard torn mattresses. The increased workload affects more than clinical services- it also has an impact on services ablution facilities, toilets, water supply and power.

#### Toilets

In low resourced health facilities, patient toilets and sanitation facilities are of low priority and are often neglected when cleaning and maintenance programme are developed. In 2015, a WHO report highlighted that 20% of healthcare facilities globally had either no provision for toilets or were in inadequate numbers (between 1 and 3.5 toilets per facility) [27–29]. In a recent systematic review of WASH activities, the lack of cleanliness of toilets was a bigger problem than the absence of toilets; this was attributed to a lack of, or inadequate access to, clean water and had a significant negative impact on patient’s assessment of care [30]. The impact of inadequate sanitation has a direct effect on increased transmission, particularly of MDROs (gram negative) and HAIs [9, 19].

#### Misuse of hand wash sinks

Sinks used for cleaning instruments were also used by surgeons and nurses to ‘scrub’ prior to surgery [31]. The WHO guidelines on decontamination and reprocessing of medical devices [32] outlines best practices including layout of a sterile services department or decontamination unit to ensure optimal workflow and cleanliness. It is concerning that the lack of physical separation of dirty and clean areas for decontamination results in mixing of clean and dirty medical devices and linen was reported [33] clearly suggesting that sterile services required review and training. It is noteworthy that the environment for reprocessing medical devices in HI is not documented as a potential source for HAI, however in LMICs it must always be taken into account when considering SSIs particularly because of inadequate cleaning of medical devices [33].

#### Inadequate supply of potable water

Effective environmental cleanliness of healthcare settings depends on the availability of safe and sufficient water for routine sanitation [21]. In a recent WHO/ UNICEF survey, 25% of health care facilities globally, lacked the basic services, (mainly located in LMICs) where constant water supply was either absent or limited [34]. The first- global WASH assessment of health care facilities was run in 54 LMICs and surveyed 66,101 facilities where 38% of them lacked access even to rudimentary levels of WASH. Large disparities also existed within countries and among facility types [35]. The Global Analysis and Assessment of Sanitation and Drinking water (GLAAS) survey report [36] revealed that only 25% of 86 countries that responded had a fully implemented plan or policy for drinking-water and sanitation in health care facilities. It was noted that countries with national plans had a greater proportion of facilities with water services, suggesting that national policies were essential for improving services. The absence of basic services has an adverse impact on the implementation of WASH services and IPC measures including environmental cleaning. Many outbreaks of HAIs in LMICs have been linked to contaminated water used during patient care especially maternal and child health and the cleaning of medical devices [37]. In India, Zambia and Tanzania, the lack of WASH facilities in delivery rooms has been commonly cited as a reason for preferring home delivery [30, 38, 39]. LMICs are increasingly encouraged to invest in water safety and ensure provision of clean water especially to healthcare facilities [9, 34]. Where there is an inadequate or lack of water supply, boreholes may be sunk, or large reservoirs constructed to collect, and store rainwater and effective biocides used to clean the water storage tanks. It is important that such water is treated and tested regularly for microbiological growth at points-of-use to ensure that the supplied water is safe [40].

#### Hospital waste management

There are significant gaps in levels of compliance with recommended best practice for sustainable waste management and environmental cleanliness [41]. The lack of source segregation at point of use, colour coding up to unsafe transportation in open trucks to the end point disposal remains a major hazard and can lead to environmental contamination and occupational health dangers in healthcare settings. Final disposal of waste remains limited to crude incineration, open dumps and landfills with the potential for environmental pollution [42–45].

#### Environmental cleaning

**Fig. 1:**
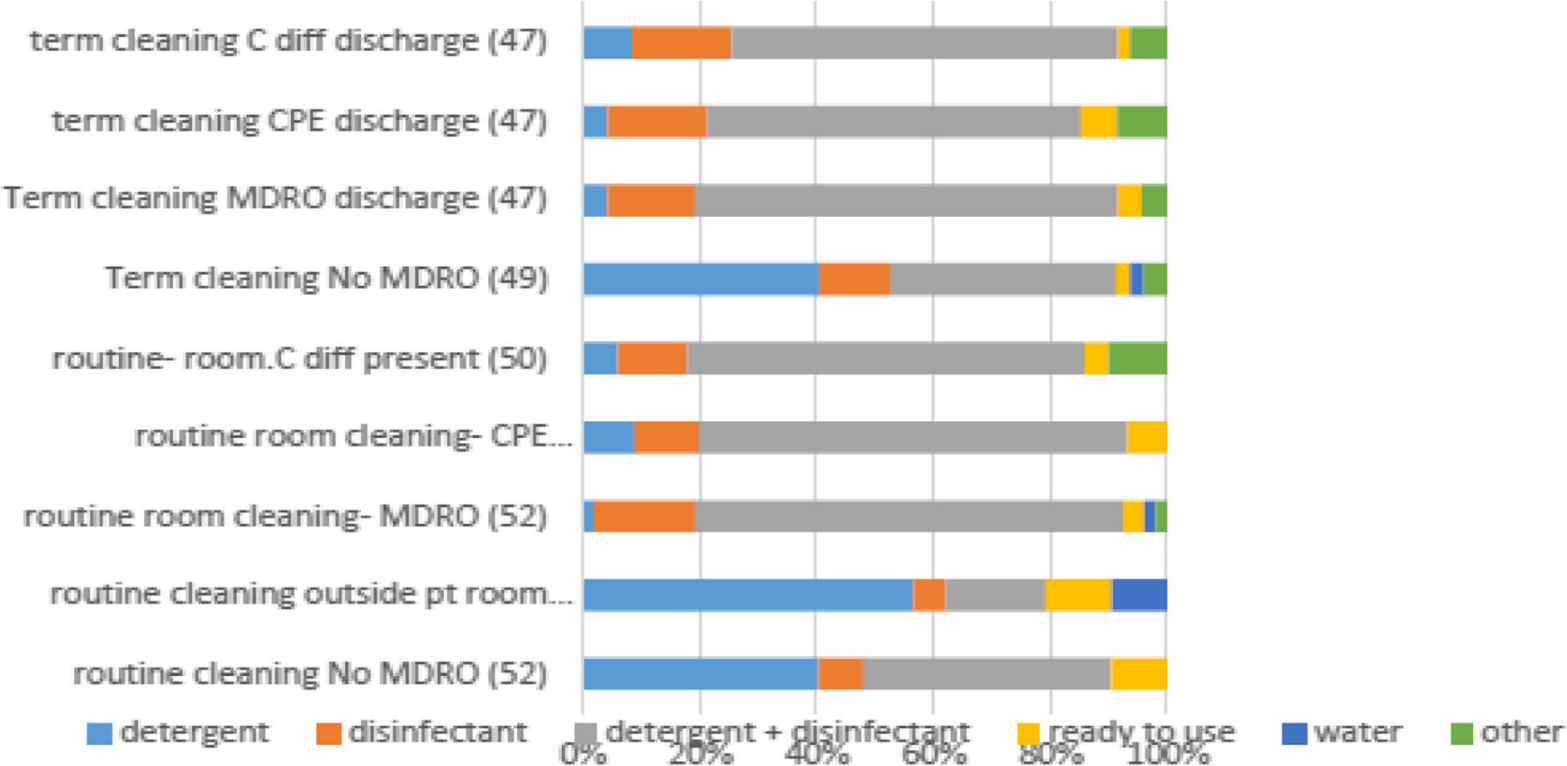
Routine and Terminal cleaning methods from ICAN survey (2016)

In 2018 the International Society of Antimicrobial Chemotherapy (ISAC) published the results of an international survey with 110 replies from 23 countries [46]. Despite the existence of policies and training in 90, and 70% respectively methodology varied across the survey. ICAN, in collaboration with ISAC extended the survey to Africa. Seventy one replies were received from 15 African countries. Of interest were the differences in cleaning methods, chemicals and frequency (Fig. 1). Routine cleaning was either with water and detergent or disinfectant, while disinfectants were mainly used for terminal cleaning. Most (90%) of the microorganisms present in visible dirt can be eliminated by routine cleaning using damp dusting, mopping with clean water and a detergent therefore routine disinfection is not indicated. Housekeeping staff must be trained regarding appropriate cleaning agents and the correct in use dilutions to avoid over-dilution of disinfectant solutions in a bid to stretch their use [47]. The newer methods proposed as ‘non-touch’ (automated) decontamination technologies, for terminal cleaning, such as vaporized hydrogen peroxide or ultraviolet light are either unavailable or unaffordable in most LMICs.

### Human capacity development

Often there is no clear job description for healthcare workers and therefore there is a lack of accountability - an essential part of good practice in healthcare. Healthcare workers continue to lack access to basic, practical education, information and effective training in IPC best practice especially for environmental hygiene, cleanliness and decontamination and there are few opportunities for learning [48, 49]. In many facilities in Africa only 1–12% of hospital staff responsible for sterile processing and standard environmental cleaning are trained [33, 50].

Training of cleaning and housekeeper staff, whether in-house or outsourced, must be according to evidence-based guidelines and standard operating procedures laid out by the healthcare facility as part of the role of the IPC Committee. The training should be contextually appropriate (local languages and dialect) and use various methods (posters, videos). The training must competency-based which will require an examination and demonstration of correct practices [21, 51, 52]. Equally, the trainers should be qualified, and periodic proficiency evaluations of trainees should be undertaken to sustain effectiveness. ICAN is committed to improving IPC practices through structured training and knowledge transfer in Africa as part of its *“From Cape to Cairo* “education programme. Training for all levels of staff including both clinical and non-clinical staff, has to be competency based. Qualification for IPC professionals requires knowledge at Postgraduate Diploma level so that IPC support to healthcare facilities is maximally effective including training and mentoring of cleaning staff.

### Clinical and corporate governance

Unlike in high income countries, there is a paucity of reliable data to support cost effectiveness and impact studies of a clean environment in LMICs. This is due to limited publications, record-keeping and inadequacies in the follow-up of patients [21, 53].. However, the WHO and more recently CDC/ICAN (2019) has published several best practice documents, on environmental cleaning and disinfection.(10) Further the WHO has tools on surveillance of WASH infrastructures for cleaner environments with a focus on LMIC countries, which can be adopted and adapted to individual country needs [9]. Despite this, these guidelines are not consistently adhered to in many countries [21, 54]. Where they do exist, implementing practices for environmental decontamination has been ineffective. This is due in part to a lack of standardized protocols and efficient processes for evaluation [54]. In fact, national IPC polices and guidelines that are based on practical, low-cost, evidence-based strategies are yet to be developed for many LMICs [11, 54, 55].

### Challenges implementing effective processes, procedures and protocols

Implementing effective processes, procedures and protocols rests on a foundation of an effective organisational structure, availability of supplies, trained personnel and accountability. The procedures are usually concerned with general techniques, Cleaning of patient areas, terminal cleaning, management of spills of body fluids, cleaning patient-use items, cleaning toilets, critical and non-critical areas and assessments of cleanliness [10, 54–56].

In many countries both high and low income, cleaning is considered a menial job. In LMIC, these jobs are delegated to hospital attendants (lower job categories) with poorer educational levels who are usually untrained in cleaning protocols and practices resulting in poor outcomes and an increased risk of contamination [57]. Cleaners are often supervised by a nurse or cleaning supervisor who usually do not have specialised training on cleaning healthcare spaces. Standardized competency training and proper supervision including verification checks are essential and should be carried out routinely with the results being presented to the IPC Committee (if there is one) for further guidance. However, the cleaning is often not organised and written protocols and checklists are not available. This poor documentation and absence of written protocols and procedures cuts across all areas of IPC [55].

Best practices include the use of clean water and detergent for routine use [10] but where terminal cleaning is indicated after the discharge of an infectious patient, an appropriate disinfectant (either 70% alcohol without any other additions such as chlorhexidine, or 0.2–0.5% chlorine (for *C. difficile*) is to be applied on a cloth not sprayed after the surfaces have been thoroughly cleaned and dried [58]. Unavailability of appropriate materials for cleaning which are contextually appropriate is also a challenge in LMICs [44] but can be overcome by training cleaning and housekeeping staff on best practices [47]. Assessment of cleanliness is usually done visually in LMICs, a method that is no longer recommended [19]. Simple but efficient non-microbiological methods for reliable assessment of surface decontamination such as reflective surface markers and adenosine triphosphate analysis can be used [57, 59]. Microbiological methods where available should be used in consultation with the IPC team particularly during an outbreak investigation [60].

#### Compliance with hand-hygiene procedures and protocols

Hand hygiene facilities is an essential component in preventing environmental contamination and HAI. Hand hygiene (HH) is, singularly, the most cost-effective IPC intervention yet suffers from a lack of practice and provision of adequate water supply. Alcohol based hand rub (ABHR) for hand hygiene has taken over from routine hand washing (except when hands are visibly contaminated) thereby reducing the need for water. In Nigeria, healthcare workers at a university teaching hospital paid attention to hand hygiene only when it appeared that there was an observable threat to their wellbeing [61]. Poor compliance with basic hand hygiene and hospital cleaning practices contribute to environmental contamination and can be addressed through provision of alcohol hand rub, facilities for handwash at convenient distances, reminders in the workplace, education and training [22].

### Funding of environmental cleaning programme

More than 52% of healthcare financing in Africa relies heavily on personal payments for health services and is largely augmented by financial assistance from bilateral and multilateral donors [62]. Budgetary allocation for the prevention of HAIs do not exist [63] and modern environmental decontamination interventions are not regarded as urgent in resource-poor settings. A lack of political will is a major impediment to encourage government funding and involvement in sanitation and hygiene programs [64], especially if there is a lack of accountability and motivation around funding sanitation and hygiene programs [65]. In order to address the issue of inadequate funding particularly for environmental cleanliness in hospital facilities, it is important to promote and encourage the objectives of WASH in government planning and budgeting [35]. This can be achieved through lobbying government officials as well as other relevant stakeholders [66]. Although globally, governments have increased allocation of national funds and spending for universal WASH in schools and health care facilities [34, 67], there remain significant gaps between plans and available budgets within many LMICs, with 80% of countries reporting that they lack sufficient funds to dedicate for WASH [68]. The commitment made by Heads of State in the African Union to allocate 15% of their respective national budgets to health at the Abuja Declaration of 2001 [69] is yet to be implemented. Limited funding is an enduring challenge in LMICs with a high dependence on donors whose policies may not always be in alignment with the national priorities. Systems need to be established to ensure the judicious, conscientious and rational use of funds allocated for improvements and new developments in environmental decontamination [70]. It is imperative for LMICs to explore new and sustainable ways of health funding.

## Discussion

Evidence supports the importance of a clean environment essential for driving down HAIs. The burden of environmental contamination is greater in low-resource settings and the challenges outlined in this review involve structure, process and outcome that exist at various system levels.

The lack of clinical governance, effective implementation strategies, guidelines and standards and most particularly funding leaves issues of environmental sanitation and hygiene low on the priority list. Where funds are available these are usually redirected for other purposes. Sadly, the cost of revising the current infrastructure in most LMICs is formidable and therefore has been side-lined or put on the back burner. Finally, the processes and administrative commitment to tackle and improve the WASH infrastructure is not in place. This could be either due to a lack of knowledge, financial resources or simply because it is of lower priority. The importance of the environment in the transmission of HAI is well documented. It is also evident that it is not just an LMIC concern and that major outbreaks with MDRO also occur in high income countries. So, the question is, where does this accountability lie? And how does it translate to practice? The answer lies within the healthcare systems. The challenges relating to environmental contamination in healthcare settings for LMICs are related to weak health systems that have been sustained by the lack of political will that manifests as inadequate governance, low accountability, inadequate allocation of resources, paucity of data relevant to decision-making, inadequately trained human resources, and inadequate infrastructure [4].

The challenges of environmental contamination therefore need to be addressed using a multi modal approach [71] and requires input from industries not traditionally associated with health-care delivery and service provision. The multimodal strategy builds on theories of change and is made up of several components that should be implemented in an integrated way to improve outcome taking into account the local context. This will include components that address the infrastructure, the supplies, the knowledge, attitude and practice of personnel, the workplace governance and the plans for sustainability and quality assurance.

The solutions will therefore include changes to infrastructure such as covering porous surfaces with hardwearing non- porous linoleum which can be found in many markets in developing countries and are relatively inexpensive, raising awareness of administrators about the dangers of unclean environments, training of all stakeholders involved in the cleaning process and governance including end users. Increased awareness of the importance of environmental hygiene by the facility administration may lead to improved organization of processes, support for staff and increased allocation of funds.

What is often required to start the process of change is a committed individual, a champion. This individual needs to be not only knowledgeable about the task but also the healthcare system within which he works. He must be passionate enough to bring his creativity to bear on surmounting the many challenges that are present in the healthcare space. The champion must be able to motivate others to commit to the time, activities and effort required to change their circumstances. Champions can be described as sociological citizens in the workplace bringing their diligence to bear on improving outcomes [72]. In some settings it has been the infection control officer, a link-nurse or the environmental supervisor.

The need to adopt appropriate technology and country-specific strategies in order to achieve the common goal of preventing environmental contamination, HAI and AMR is also recommended. Strategic goals that support the implementation of respective national standards in ways that reflect local and country-specific conditions should be established [73]. Implementation tools to help countries achieve their goals are recommended and have been developed by the WHO [74]. Policies will provide a framework for configuration, utilization and quality management strategies to address the various challenges resulting from environmental contamination. Leadership is crucial and an increase in political awareness can lead to the much needed changes to improve outcomes [51] such as more trained IPC professionals including cleaning staff that are required to drive the process of reducing environmental contamination by working with other healthcare workers to develop policies, set standards and ensure effective implementation of processes, practices and training programs.

## Conclusion

The challenges encountered in the control of environmental contamination in Low and middle income countries though mostly similar to those in high income countries are significantly more pronounced as a result of the poor infrastructure, lack of prioritisation, inadequate numbers of trained personnel and poor funding. The evidence showing the role of a clean environment in reducing the rates of Healthcare associated infections is compelling and has been encapsulated in the core components of an effective infection control program. It is evident that the challenges identified are already recognised by the countries and in many, some effort is already being made. However, more needs to be done and requires committed leadership to provide the framework for and drive the change to provide a safe environment for healthcare.

## Data Availability

“Data sharing not applicable to this article as no datasets were generated or analysed during the current study.
